# Measuring Urban Vibrancy of Residential Communities Using Big Crowdsourced Geotagged Data

**DOI:** 10.3389/fdata.2021.690970

**Published:** 2021-06-10

**Authors:** Pengyang Wang, Kunpeng Liu, Dongjie Wang, Yanjie Fu

**Affiliations:** Computer Science Department, University of Central Florida, Orlando, FL, United States

**Keywords:** urban vibrancy, spatiotemporal data mining, urban computing, Big Crowdsourced Geotagged Data mining, learning-to- rank

## Abstract

The pervasiveness of mobile and sensing technologies today has facilitated the creation of Big Crowdsourced Geotagged Data (BCGD) from individual users in real time and at different locations in the city. Such ubiquitous user-generated data allow us to infer various patterns of human behavior, which helps us understand the interactions between humans and cities. In this article, we aim to analyze BCGD, including mobile consumption check-ins, urban geography data, and human mobility data, to learn a model that can unveil the impact of urban geography and human mobility on the vibrancy of residential communities. Vibrant communities are defined as places that show diverse and frequent consumer activities. To effectively identify such vibrant communities, we propose a supervised data mining system to learn and mimic the unique spatial configuration patterns and social interaction patterns of vibrant communities using urban geography and human mobility data. Specifically, to prepare the benchmark vibrancy scores of communities for training, we first propose a fused scoring method by fusing the frequency and the diversity of consumer activities using mobile check-in data. Besides, we define and extract the features of spatial configuration and social interaction for each community by mining urban geography and human mobility data. In addition, we strategically combine a pairwise ranking objective with a sparsity regularization to learn a predictor of community vibrancy. And we develop an effective solution for the optimization problem. Finally, our experiment is instantiated on BCGD including real estate, point of interests, taxi and bus GPS trajectories, and mobile check-ins in Beijing. The experimental results demonstrate the competitive performances of both the extracted features and the proposed model. Our results suggest that a structurally diverse community usually shows higher social interaction and better business performance, and incompatible land uses may decrease the vibrancy of a community. Our studies demonstrate the potential of how to best make use of BCGD to create local economic matrices and sustain urban vibrancy in a fast, cheap, and meaningful way.

## Introduction

Vibrant residential communities (vibrant communities for short) are defined as places that show diverse and frequent consumer activities. Vibrant communities usually have the following features: permeability, vitality, variety, accessibility, identity, and legibility. Developing vibrant communities is very beneficial for both social good and business good. For instance, vibrant communities can attract talented younger workers, high-tech entrepreneurs, and cutting-edge firms, as well as foster intensive social interactions, productivity, and creative activities. Thereby, understanding urban vibrancy can help 1) contribute to economic growth; 2) enhance public security; and 3) improve environmental, fiscal, and social outcomes. For example, when hunting for a business site, entrepreneurs should consider the surrounding community, whether it is welcoming and attractive for business activities (Church and Murray, 2009). By studying the urban vibrancy patterns of communities, we can make better decisions and suggestion for business site selection, to ensure successful business.

However, it is traditionally challenging to develop vibrant communities because there is not a clear answer to the following question: “what kind of communities tend to have higher vibrancy?” In prior literature, researchers have conducted conceptual and empirical studies about vibrant communities in the fields of urban planning and social science. For example, Glaeser *et al.* pointed out that vibrant communities depend on the demand for urban *density* (Glaeser et al., 2001). Couture *et al.* found people are willing to pay higher rents and transportation costs for vibrant places (Couture, 2013). Farber *et al.* found that vibrant communities are associated with spatial concentration of residents and diversity of products and services (Farber and Li, 2013). Malizia *et al.* found that vibrant communities are usually compact, dense, and accessible with diverse land uses (Malizia and Song, 2014). Neutens *et al.* found that high-density and mixed land uses can benefit quality social interactions and enhance community vibrancy (Neutens et al., 2013). Dougal *et al.* argued urban vibrancy can be reflected by dynamic human-dependent factors (e.g., highly talented workers) that vary over time (Dougal et al., 2015). However, all these studies only provide conceptual understanding on one or two aspects of the community vibrancy.

In order to provide a comprehensive understanding of various aspects that contribute to the community vibrancy, we propose a big data–driven approach which is the first time to systematically study the measurements and patterns of vibrant communities. Specifically, we take advantage of the large-volume and ubiquitous user-generated data collected from diverse sources, for example, buildings, vehicles, human, sensors, and devices, in real time and at different locations in the city. Such Big Crowdsourced Geotagged Data (BCGD) allow us to infer various patterns of human behavior and understand the interactions between humans and cities. If properly analyzed, these data can be a rich source of intelligence to discover and mimic the unique spatial and mobility patterns of vibrant communities.

However, due to the variety and veracity nature of big data, it is very challenging to analyze BCGD. To make the analysis effective and efficient, we propose to focus the community vibrancy analysis on two perspectives: 1) spatial configuration and 2) social interaction. First, the spatial configuration of a community is empirically defined as the physical characteristics that make up built-up areas, such as bus systems, subway systems, road networks, and landmarks, as well as corresponding locations, numbers, and mutual distances. Prior literature has developed empirical evidence that suggests the significant impact of spatial configuration on community vibrancy (Song and Knaap, 2004; Koster and Rouwendal, 2012; Loehr, 2013). However, it is not a trivial task to quantify the spatial configuration of communities. Particularly, we need to construct effective variables (i.e., features) from static urban geography data (e.g., landmarks, public transportation data, and road network data), in order to capture the compatible dimensions of spatial structure, as well as the corresponding portfolios and geographic allocations of these dimensions within a community. Second, from the perspective of social interaction, there are some preliminary studies (Farber et al., 2013, 2014; Farber and Li, 2013; Neutens et al., 2013) about measuring general social interactions using human mobility data. Unfortunately, since human mobility data are mostly in a form of trajectories or footprints, typically represented by a sequence of GPS location points, such data are lack of semantically rich information, which makes the task of profiling social interactions within and across communities very challenging. Therefore, we propose to augment and enrich the semantic information of human mobility data in order to analyze intercommunity and intra-community social interaction. In summary, we propose to analyze and extract the features of spatial configuration from urban geography data and the features of social interaction from human mobility to spot highly vibrant communities, which will be formulated as a ranking-based data mining task next.

Although a lot of features may be extracted from a variety of data sources, these extracted vibrancy-related features are often correlated and redundant. The feature redundancy can result in poor generalization performance. In reality, a small number of good features are usually sufficient to represent the patterns of vibrant communities and facilitate accurate prediction of spotting vibrant communities. Conventional methods usually use a two-step paradigm, which is basically to first select a feature subset and then learn a ranking model based on the selected features. However, the selected feature subset may not be optimal for ranking because the two steps are modeled separately. As revealed by many machine learning researchers, the presumption of the sparsity-regularized classification models is that only a subset of features are significant for prediction; that is, the coefficients of nonsignificant features will be very small and close to zero in the learned classification model. Therefore, we propose to combine sparsity regularization and ranking objective in a unified model to help us identify the optimal feature subset for spotting vibrant communities.

To summarize, in this article, we conduct a systematic study on the measurements, patterns, and modeling of urban vibrancy. Specifically, the following are our main contributions: 1) We start with defining a fused scoring method based on F-measure to quantify the urban vibrancy of communities. 2) We mine the features of spatial configuration from static urban geography data and the features of social interactions from dynamic human mobility data. 3) Given the obtained features, we develop a novel model to learn the patterns of vibrant communities, by combining pairwise ranking objective and sparsity regularization in a unified probabilistic framework, which is greatly enhanced by simultaneously conducting feature selection and maximizing ranking accuracy. 4) Finally, we conduct comprehensive performance evaluations for the feature sets and models with large-scale real-world data, and the experimental results demonstrate the competitive performance of our method with respect to different validation metrics.

## Problem Statements and Framework Overview

In this section, we first introduce the important definitions and formulate the problem. After that, we provide an overview of the proposed analytic framework.

### Definitions and Problem Formulation

**FIGURE 1:**
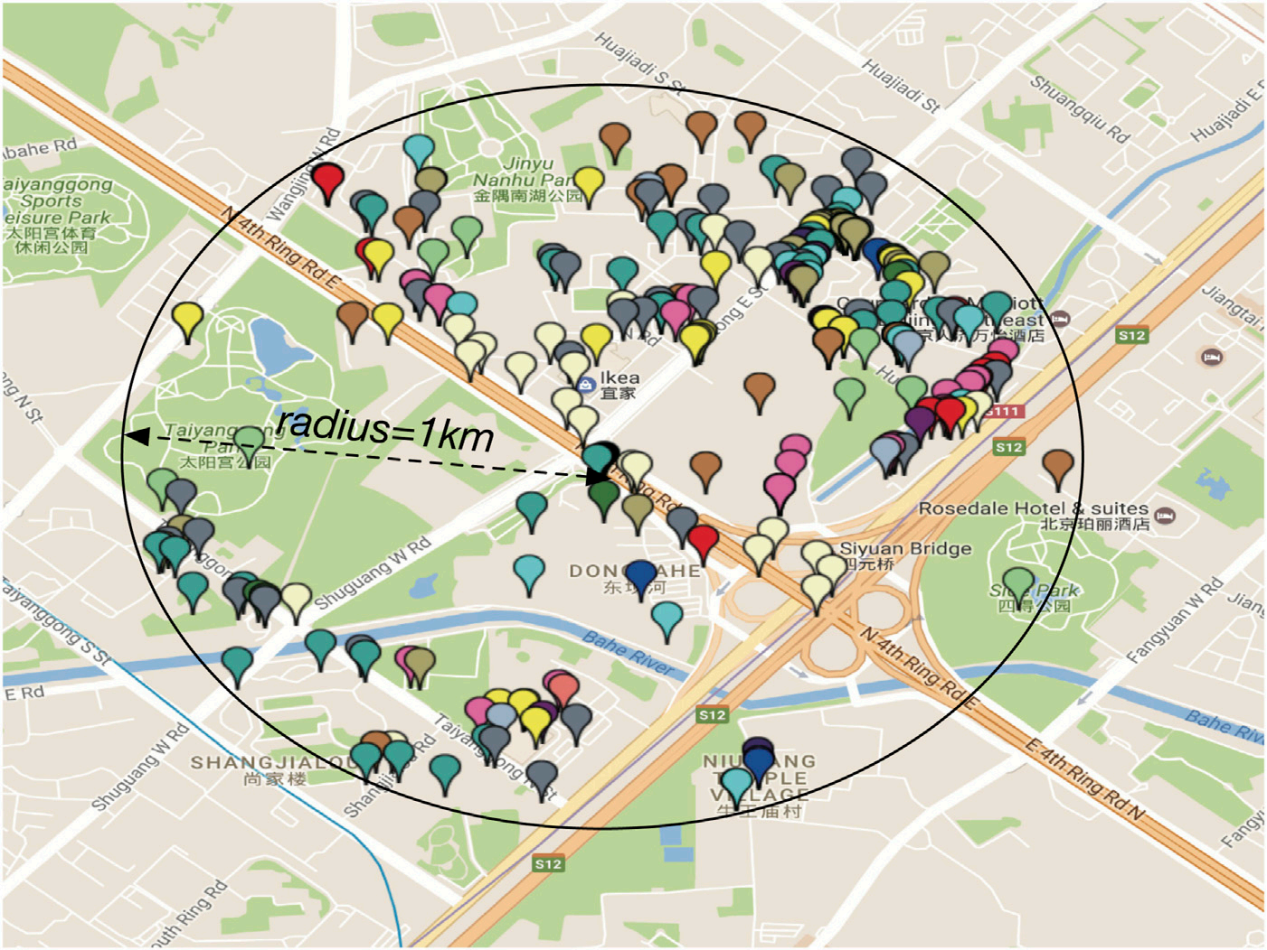
Example of a residential community.

Residential community: A residential community consists of a location (i.e., latitude and longitude) of a residential complex and a neighborhood area (e.g., a circle with radius of 1 *km*). A residential complex often includes one or multiple apartment buildings in urban areas. There are a variety of point of interests (POIs) in the neighborhood area, providing many services to people. Figure 1 shows an example of a residential community.

Problem definition: Formally, given a set of *I* residential communities $X=\left\{x_{1},x_{2},\ldots ,x_{I}\right\}$, the goal of our problem is to rank them in a descending order according to their vibrancy scores $Y=\left\{y_{1},y_{2},\ldots ,y_{I}\right\}$. BCGD such as point of interest data, human mobility data, and mobile consumption check-in data have encoded the unique spatial and social patterns of residential communities, and thus can be used to identify vibrant communities by exploiting a data-driven analytics-enabled strategy. Essentially, there are three major tasks: 1) Developing empirical and measurable metric to quantify the vibrancy scores of residential communities; 2) quantifying the patterns of spatial configuration and social interaction within and across residential communities; and 3) learning to spot highly vibrant communities with the spatial and social patterns of communities.

### Framework Overview

The focus of this article is to develop a data mining approach for spotting vibrant residential communities. In the pursuit of this general aim, we have three specific tasks: measurement, patterns, and modeling.

• In researching measurements, we aim to develop an empirical metric to measure community vibrancy using a data-driven strategy. While urban vibrancy is difficult to be observed, BCGD provide a potential to circumvent this problem. To quantify vibrancy empirically, we make use of novel mobile consumption check-in data and propose an unsupervised fused scoring method to quantify the vibrancy score of each community.
• In researching patterns, we aim to discover the patterns of community vibrancy. We extract various contextual features from two perspectives: spatial configuration and social interactions. The spatial configuration features are extracted from the urban geography data including public transportation, road networks, and POIs; the social interaction features are extracted from the human mobility data including bus GPS data, taxi GPS data, and smartphone GPS data.
• In researching patterns, to make full use of all relevant features, we develop a sparse learning-to-rank approach for spotting vibrant communities.

**FIGURE 2:**
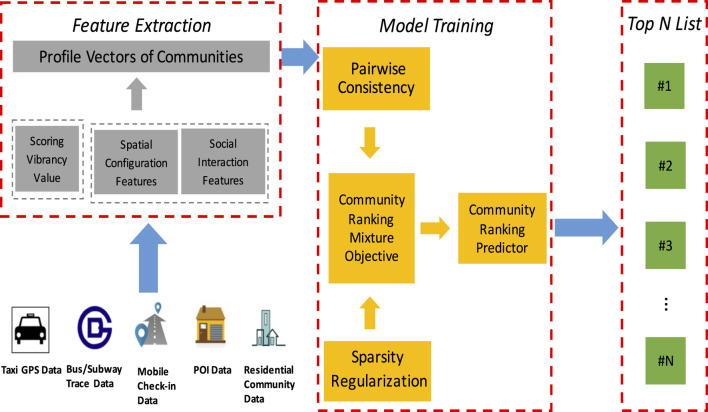
Overview of our framework.

Figure 2 shows the overview of the proposed analytic framework.

## An Empirical Metric for Estimating Community Vibrancy

In prior literature, researchers have found that the vibrancy score of a residential community can be reflected by consumer activities from two perspectives: density and diversity of consumer activities (Talen, 1999; Glaeser et al., 2001; Couture, 2013; Farber and Li, 2013; Malizia and Song, 2014). Here, “density” can be explained by the fact that if a large number of consumers are willing to pay higher transportation costs to visit a place, and to spend more time to consume in that place, the place is likely to be vibrant. A high “diversity” of consumer activities indicates that this place can meet a variety of consumption needs and help consumers carry out different outdoor activities in a single place within a walking distance. In other words, consumers do not have to visit other places and can complete a variety of activities in a single place.

To capture vibrancy empirically, we make use of a novel geotagged user consumption check-in data shared in location-based social networks (LBSNs). A check-in event contains the information of a mobile user’s destination POI and consumption activity type, which connects user profiles, POI locations, and outdoor activities with measurable density and diversity. The presumption is that urban vibrancy increases the density and diversity of consumer activities and POIs in a place. In other words, urban vibrancy promotes the probability that mobile users check into a place, enhances the diversity of urban functions, and improves social interactions and centralization across different categories of outdoor activities. With this presumption, urban vibrancy, even though not observed directly, can be identified by strategically fusing the observable densities and diversities of mobile check-ins over various activity categories, for example, home, work, date, dinning, travel, transportation, shopping, and entertainment. Specifically, urban vibrancy can be quantified by mathematically giving a vibrancy score using a fused scoring framework. We propose to proceed with three steps: 1) measuring the density of consumer activities, 2) measuring the diversity of consumer activities, and 3) fused scoring.

 1) Measuring the density of consumer activities.

We propose to extract the density of consumer activities in communities. For each residential community, we count the total number of mobile consumption check-in events (#) as an estimation of the density of consumer activities, denoted by fre=#.

 2) Measuring the diversity of consumer activities.

To estimate the diversity of consumer activities in a community, we count the numbers of mobile check-in events with respect to different POI categories, denoted by ${\left\{{\#}_{c}\right\}}_{c=1}^{\mathbb{C}}$, where *c* represents the *c*-th POI category and ℂ denotes the number of POI categories. We compute the diversity of consumer activities by exploiting the definition of entropy

$$\text{div}=\overset{\mathbb{C}}{\underset{c=1}{\sum }}{\#}_{c}\text{log}{\#}_{c}.$$ (1)

 3) Fused scoring.

After extracting and normalizing both density and diversity, we use the F-1 score

$$\text{vibrancy}=\frac{2\;\;*\;\text{fre}\;\;*\;\;\text{div}}{\text{fre}\;\;+\;\;\text{div}}$$ (2)

to fuse both density and diversity into a single score. The score extracted from consumer activities can empirically measure the vibrancy of a community.

## Discovering Patterns of Vibrant Communities

We now proceed to introduce discriminative features to describe and quantify the patterns of vibrant communities. Specifically, we categorize the features into two categories:

• *The features of spatial configurations*, which can be extracted from urban geography data, such as public transportation, road networks, and POIs.
• *The features of social interactions*, which can be extracted from human mobility data (taxi GPS data, bus GPS data, etc.) within and across communities.

### Features of Spatial Configuration

The spatial configuration of a community is a three-element tuple, including 1) the compatible dimensions of the spatial configuration, such as shopping, living service, education, and transportation buildings that serve important urban functions; 2) the portfolio of these compatible dimensions, such as frequency, density, and diversity of different POIs; and 3) the geographic allocation of these compatible dimensions, such as distances to different POIs. The recent study by Evans et al. (2007) implied that urban environmental elements combine to determine the quality of life in higher density and mixed-use locations. Moreover, the study by Yue et al. (2017) showed that POI diversity contributes significantly to improving neighborhood vibrancy. Therefore, we extract 1) density of POIs, 2) diversity of POIs, and 3) accessibility of transportation as features for each community $c_{i}$, in which there are a set of POIs, denoted by $P=\left\{p:p\in c_{i}\&p\in P\right\}$, where *p* is a POI.

 1) Density of POIs.

After studying large-scale residential community data, mobile check-in data, and POI data, we found that the vibrancy level of a place depends on the density of POIs in the same area. Intuitively, the more POIs in a community, the more likely the community could meet a visitor’s various needs, such as dating, shopping, and watching movies. Therefore, we exploit the density of POIs as a feature. Specifically, for each community $c_{i}$, we can count the POI number of each POI category $\phi_{k}$. POI categories are defined based on their functions, such as shopping, sports, and education. Formally, we have

$$num_{c_{i}}^{\phi_{k}}=\sum I\left\{p\in c_{i}\&p\in P\&p\in \phi_{k}\right\},$$ (3)

where *I* denotes the numbers of POIs. We note that given that the radius of a community is the same, the density depends only on the number of POIs they include.

 2) Diversity of POIs.

To assess the influence of the spatial heterogeneity of community functionalities on the vibrancy of a community, we apply the entropy measure to describe the diversity of POIs for a community. For each community $c_{i}$, we calculate the diversity $\eta_{i}$ as follows:

$$\eta_{i}=-\underset{k}{\sum }\frac{num_{c_{i}}^{\phi_{k}}}{\sum_{k}num_{c_{i}}^{\phi_{k}}}\text{log}\frac{num_{c_{i}}^{\phi_{k}}}{\sum_{k}num_{c_{i}}^{\phi_{k}}}.$$ (4)

According to the definition, the larger the entropy is, the higher diversity the community has. Be sure to notice that the diversity of POIs has correlation with the diversity of user consumption activities in the measurement section of community vibrancy, but they are two different concepts. The diversity of POIs represents the spatial configuration and geographic allocation of a community; the diversity of user consumption activities denotes a quantitative aspect of human dynamic behavior.

 3) Accessibility.

We refer accessibility to the degree of convenience that consumers can visit a community. For example, street connectivity, higher bus stop density, and greater nonmotorized access promote the possibility of human mobility and influence the transportation mode choice (Khan et al., 2014); different effects of spatial accessibility vary among different trip purposes (Zhang, 2005); and users in different gender and youth groups show different mobility patterns in rural and suburban areas (Collins et al., 2012). Generally, public transportation facilities and the quality of the road network are two basic factors that influence the accessibility.

$$num_{c_{i}}^{b}=\sum I\left\{b\in B\&b\in c_{i}\right\},$$ (5)

where *I* denotes the numbers of bus stops, *B* denotes the bus stop set, and *b* denotes a bus stop. Besides, we also calculate the minimum distance from POIs to the bus stops as $\zeta_{c_{i}}^{b}$:

$$\zeta_{c_{i}}^{b}=\underset{p\in c_{i}\&b\in c_{i}}{\text{min}}dist\left(p,b\right),$$ (6)

where dist(p,b) denotes the distance between a POI *p* and a bus station *b*. Similarly, for subway stations, we calculate the number of subway stations:

$$num_{c_{i}}^{s}=\sum I\left\{s\in S\&s\in c_{i}\right\},$$ (7)

where *S* denotes the subway station set and *s* denotes a subway station. And the minimum distance from POIs to the subway stations is denoted as $\zeta_{c_{i}}^{s}$:

$$\zeta_{c_{i}}^{s}=\underset{p\in c_{i}\&s\in c_{i}}{\text{min}}dist\left(p,s\right),$$ (8)

where dist(p,s) denotes the distance between a POI *p* and a subway station *s*.

1. Public transportation facilities. There are two major types of public transportation—bus and subway, in most big cities. Therefore, we define and extract some important properties of *bus stops* and *subway stations.* For each community $c_{i}$, we calculate the number of bus stations:
2. The quality of the road network. Intuitively, in an urban area, if a community has more intersections of road networks, consumers can access taxis or enter road network systems by private cars more easily. Also, if a community with the same radius has longer roads and highways, the density of road networks is higher. Therefore, we calculate *the number of intersections of roads* (denoted as $num_{c_{i}}^{\tau }$) and *the density of road networks* (denoted as $v_{i}$) to measure the quality of road networks.

For each community $c_{i}$, the number of intersections of roads $num_{c_{i}}^{\tau }$ can be calculated as

$$num_{c_{i}}^{\tau }=\underset{\tau }{\sum }I\tau \in c_{i},$$ (9)

where τ denotes an intersection in the road networks, and the density of road networks $v_{i}$ can be calculated as

$$v_{i}=\underset{\tau_{k}\in c_{i}\&\tau_{l}\in c_{i}}{\sum }dist\left(\tau_{k},\tau_{l}\right),$$ (10)

where $dist\left(\tau_{k},\tau_{l}\right)$ denotes the distance between two intersections $\tau_{k}$ and $\tau_{l}$.

### Features of Social Interactions

Social interactions within and across communities can be observed and estimated from people’s movements. In general, human mobility encodes two types of social interactions:

• *The interactions between users and users*: A mobile user moves from one community to another community and stays in the destination for a certain time. During this time period, the mobile user is highly likely to meet and speak to other mobile users, particularly for the trip purpose of dating, entertainment, and dinning.
• *The interactions between users and places*: Mobile users inevitably have to interact with a variety of POIs to complete activities with respect to different trip purposes, such as working, shopping, dining, and entertainment.

As a result, we extract social interaction features from the human mobility data based on the following three perspectives: *(i) mobility flow, (ii) range, and (iii) average speed*.

 1) Mobility flow.

Taking a community as an example, we can observe movements that leave a community, arrive at a community, and transit within a community. Based on the above observations, all movements can be segmented into three types: *1) inflow* (corresponding to arriving human mobility), *2) outflow* (corresponding to leaving human mobility), and *3) intra-flow* (corresponding to human mobility within communities). In BCGD, a movement trajectory $tr_{k}$ can be represented as a four-element tuple $\left(O_{k},t_{O_{k}},D_{k},t_{D_{k}}\right)$, where $O_{k}$ denotes the original point, $t_{O_{k}}$ denotes the start time, $D_{k}$ denotes the destination point, and $t_{D_{k}}$ denotes the end time.

$$inflow_{c_{i}}=\sum_{k}I\left\{O_{k}\notin c_{i},D_{k}\in c_{i}\right\},$$ (11)

where *I* denotes the number of trajectories and $inflow_{c_{i}}$ denotes the inflow volume of the community $c_{i}$.

$$outflow_{c_{i}}=\sum_{i}I\left\{O_{k}\in c_{i},D_{k}\notin c_{i}\right\},$$ (12)

where *I* denotes the numbers of trajectories and $outflow_{c_{i}}$ denotes the outflow volume of the community $c_{i}$.

$$intra-flow_{c_{i}}=\sum_{i}I\left\{O_{k}\in c_{i},D_{k}\in c_{i}\right\},$$ (13)

where *I* denotes the number of trajectories and $intra-flow_{c_{i}}$ denotes the intra-flow volume of the community $c_{i}$.

 2) Range.

1. Inflow interaction. Inflow is defined as movements that people come to visit the community $c_{i}$ from other communities. Therefore, the volume of inflow can be calculated as
2. Outflow interaction. Outflow is defined as movements that people leave the community $c_{i}$. Therefore, the volume of outflow can be calculated as
3. Intra-flow interaction. Intra-flow is defined as movements that are inside of the community $c_{i}$. Therefore, the volume of intra-flow can be calculated as

We check the maximum commute distance of taxis to the community to represent the range of social interactions. For a community $c_{i}$, we calculate the range of interaction $\lambda_{i}$ as

$$\lambda_{i}=\underset{\left(O_{k},t_{O_{k}},D_{k},t_{D_{k}}\right)\in T^{taxi}}{\text{max}}dist\left(O_{k},D_{k}\right),$$ (14)

where $T^{taxi}$ denotes taxi trajectories and $dist\left(O_{k},D_{k}\right)$ denotes the distance between $O_{k}$ and $D_{k}$.

 3) Average speed.

The average speed of taxis on roads reflects the fluency of interactions. For a given community $c_{i}$, we calculate the average speed ${\bar{\nu }}_{i}$ as

$${\bar{\nu }}_{i}=\frac{\sum_{k}\frac{dist\left(O_{k},D_{k}\right)}{t_{D_{k}}-t_{O_{k}}}}{I\left\{\left(O_{k},t_{O_{k}},D_{k},t_{D_{k}}\right)\in T^{taxi}\right\}},$$ (15)

where *I* denotes the number of trajectories and *k* is legal when $\left(O_{k},t_{O_{k}},D_{k},t_{D_{k}}\right)\in T^{taxi}$.

### Feature Summary

We extract features from BCGD according to the definitions in 4.1 and 4.2. The summary of the extracted features is in Table 1. To further capture how the spatial and social features vary over community radius, we set the radius of a community as different distance values (e.g., 0.25, 0.5, 0.75, 1, 1.5, 2, 2.5, and 3 km) and extract a large number of features.

**TABLE 1:** Feature summary.

Feature type	Category	Subcategory	Denotation
Spatial configuration	Density		$num_{c_{i}}^{\phi_{k}}$
	Diversity		$\eta_{i}$
	Accessibility	Public transportation facilities	$num_{c_{i}}^{b}$
			$\zeta_{c_{i}}^{b}$
			$num_{c_{i}}^{s}$
			$\zeta_{c_{i}}^{s}$
		The quality of the road network	$num_{c_{i}}^{\tau }$
			$v_{i}$
Social interaction	Flow	Inflow	$inflow_{c_{i}}$
		Outflow	$outflow_{c_{i}}$
		Intra-flow	$intra-flow_{c_{i}}$
	Range		$\lambda_{i}$
	Average speed		${\bar{\nu }}_{i}$

## Learning to Identify the Patterns of Vibrant Communities

In this section, we present how to select the proper set of important features out of the large number of features obtained from the previous step. We propose a model to spot highly vibrant communities by combining pairwise learning to ranking and sparsity regularization.

### Model Description

Since many existing learning-to-rank algorithms use linear rankers, we also learn a linear ranking predictor. Let $x_{i}$ denote the *M*-size vector representation of residential community $e_{i}$ with the above extracted features, $f_{i}$ denote the predicted vibrancy score, and $y_{i}$ denote the ground truth of the vibrancy score, then we have

$$f_{i}\left(x_{i};w\right)=\overset{M}{\underset{m=1}{\sum }}w_{m}x_{im}+\epsilon_{i}=w^{⊤}x_{i}+\epsilon_{i},$$ (16)

where $\epsilon_{i}$ is a zero-mean Gaussian bias with variance $\sigma^{2}$ and w is the weights of the features. In other words, $P\left(y_{i}|x_{i}\right)=N\left(y_{i}|f_{i},\sigma^{2}\right)=N\left(y_{i}|w^{⊤}x_{i},\sigma^{2}\right)$, where N represents the normal distribution.

### Objective Function

While these features indeed capture the spatial configurations and social interactions of residential communities to be ranked, they are often intercorrelated and redundant. These possible confounders lead to poor generalization performance. To address this issue, we adopt a strategy which simultaneously conducts the feature selection while maximizing the ranking accuracy. Since the pairwise ranking strategy is more effective than the listwise ranking strategy, we combine a pairwise ranking objective and a sparsity regularization term in a unified probabilistic modeling framework.

Next, we introduce how to derive the objective for collectively spotting highly vibrant communities and selecting features. Let us denote all parameters by $\text{Ψ}=\left\{w,\beta^{2}\right\}$, which are the parameters of the community ranker (we will introduce $\beta^{2}$ in the following); the hyperparamters by $\text{Ω}=\left\{a,b,\sigma^{2}\right\}$, which are the parameters of sparsity regularization; and the observed data by D={Y,Π}, where *Y* and Π are the community vibrancy scores and ranks of *I* estates, respectively. For simplicity, we assume the residential communities in D are sorted and indexed in a descending order of their community vibrancy scores, which compiles a descending ranks as well. In other words, *i* is both the index and the ranking order of the given community $x_{i}$. By Bayesian inference, we have the posterior probability as

$$\begin{array}{c} Pr\left(\text{Ψ};D,\text{Ω}\right)=P\left(D|\text{Ψ},\text{Ω}\right)P\left(\text{Ψ}|\text{Ω}\right) \end{array}.$$ (17)

In Eq. 17, the term P(D|Ψ,Ω) is the likelihood of the observed data collection D, which can be explained as a joint probability of both community vibrancy scores, P(Y|Ψ,Ω), and community ranking consistency, P(Π|Ψ,Ω). Here, we treat the ranked list of communities as a directed graph, G=<V,E>, with nodes as communities and edges as pairwise ranking orders. For instance, an edge i→h representing community *i* is ranked higher than community *h*. From a generative modeling angle, the edge i→h is generated by our model through a likelihood function P(i→h). The more vibrant the community *i* is than the community *h*, the larger P(i→h) should be. On the contrary, the case, in which i→h but $f_{i}<f_{h}$, will punish P(i→h). Therefore,

$$\begin{array}{c} P\left(D|\text{Ψ},\text{Ω}\right)=P\left(Y|\text{Ψ},\text{Ω}\right)P\left(\text{Π}|\text{Ψ},\text{Ω}\right)\\ =\overset{I}{\underset{i=1}{\prod }}N\left(y_{i}|f_{i},\sigma^{2}\right)\overset{I-1}{\underset{i=1}{\prod }}\overset{I}{\underset{h=i+1}{\prod }}P\left(i\to h|\text{Ψ},\text{Ω}\right), \end{array}$$ (18)

where the generative likelihood of each edge i→h is defined as sigmoid ($f_{i}-f_{h}$):

$$P\left(i\to h\right)=\frac{1}{1+exp\left(-\left(f_{i}-f_{h}\right)\right)}.$$ (19)

Moreover, the term P(Ψ|Ω) is the prior of the parameters Ψ. Here, we introduce a sparse weight prior distribution by modifying the commonly used Gaussian prior, such that a different and separate variance parameter $\beta_{m}^{2}$ is assigned to each weight. Thus, $P\left(w|\alpha \right)=\prod_{m=1}^{M}N\left(w_{m}|0,\beta_{m}^{2}\right)$, where $\beta_{m}^{2}$ represents the variance of the corresponding parameter $w_{m}$ and $\beta^{2}={\left(\beta_{1}^{2},\ldots ,\beta_{M}^{2}\right)}^{⊤}$, each of which is treated as a random variable. Later, an inverse gamma prior distribution is further assigned to these hyperparameters, $P\left(\beta^{2}|a,b\right)=\prod_{m=1}^{M}$inverse gamma$\left(\beta_{m}^{2};a,b\right)$, where *a* and *b* are constants and are usually set close to zero. By integrating over the hyperparameters, we obtain a student-t prior for each weight, which is known to enforce sparse representations during learning by setting some feature weights to zero and avoiding overfitting:

$$\begin{array}{c} P\left(\text{Ψ}|\text{Ω}\right)=P\left(w|0,\beta^{2}\right)P\left(\beta^{2}|a,b\right)\\ =\overset{M}{\underset{m=1}{\prod }}N\left(w_{m}|0,\beta_{m}^{2}\right)\overset{M}{\underset{m=1}{\prod }}Inverse-Gamma\left(\beta_{m}^{2}|a,b\right). \end{array}$$ (20)

### Parameter Estimation

With the formulated posterior probability, the learning objective is to find the optimal estimation of the parameters Ψ that maximizes the posterior. Hence, by inferring equation 17, we can have the log of the posterior for the proposed model:

$$\begin{array}{l} ℒ\left(w,\beta^{2}|Y,\text{Π},a,b,\sigma^{2}\right)=\\ \overset{I}{\underset{i=1}{\sum }}\left[-\frac{1}{2}\text{ln}\sigma^{2}-\frac{{\left(y_{i}-f_{i}\right)}^{2}}{2\sigma^{2}}\right]+\overset{I-1}{\underset{i=1}{\sum }}\overset{I}{\underset{h=i+1}{\sum }}ln\frac{1}{1+exp\left(-\left(f_{i}-f_{h}\right)\right)}\\ +\overset{M}{\underset{m=1}{\sum }}\left[-\frac{1}{2}\text{ln}\beta_{m}^{2}-\frac{w_{m}^{2}}{2\beta_{m}^{2}}\right]+\overset{M}{\underset{m=1}{\sum }}\left[-\left(a+1\right)\text{ln}\beta_{m}^{2}-\frac{b}{\beta_{m}^{2}}\right]. \end{array}$$ (21)

We apply a gradient descent method to maximize the posterior by updating $w_{m},\beta_{m}^{2}$ through

$$w_{m}^{\left(t+1\right)}=w_{m}^{\left(t\right)}-\epsilon \frac{\partial \left(-ℒ\right)}{\partial w_{m}}$$ (22)

and

$$\beta_{m}^{2\left(t+1\right)}=\beta_{m}^{2\left(t\right)}-\epsilon \frac{\partial \left(-ℒ\right)}{\partial \beta_{m}^{2}},$$ (23)

where

$$\begin{array}{l} \frac{\partial \left(ℒ\right)}{\partial w_{m}}=\overset{I}{\underset{i=1}{\sum }}\frac{1}{\sigma^{2}}\left(y_{i}-\overset{M}{\underset{m=1}{\sum }}w_{m}\cdot x_{im}\right)x_{im}\\ +\overset{I-1}{\underset{i=1}{\sum }}\overset{I}{\underset{h=i+1}{\sum }}\frac{exp\left(-\left(f_{i}-f_{h}\right)\right)}{1+exp\left(-\left(f_{i}-f_{h}\right)\right)}\left(x_{im}-x_{hm}\right)+\frac{-w_{m}}{\beta_{m}^{2}} \end{array}$$ (24)

$$\begin{array}{c} \frac{\partial \left(ℒ\right)}{\partial \beta_{m}^{2}}=\frac{-1}{2\beta_{m}^{2}}+\frac{w_{m}^{2}}{\beta_{m}^{4}}+\frac{-\left(a+1\right)}{\beta_{m}^{2}}+\frac{b}{\beta_{m}^{4}} \end{array}.$$ (25)

## Experimental Results

We provide an empirical evaluation of the performances of the proposed method on the real-world residential community–related data.

### Data Description

We use the residential community data and crowdsourced geotagged data including bus/subway smart card data, taxi GPS traces data, POIs, and mobile check-in data in Beijing for this study.

#### Residential Community Data

Since the urban areas of big cities are usually compact due to large population, residential complexes become the major type of properties in the urban area of a city. A residential complex usually includes one or more apartment buildings. We have obtained the data of more than 3,000 Beijing residential complexes by crawling Fang.com, which is the largest real estate online system in China.

#### Crowdsourced Geotagged Data

• *Taxi GPS Data.* Taxi transits are faster and more expensive and represent an important part of human mobility. Taxi GPS sensors generate trajectory data in the form of sequences of location and time pairs. In our experiments, the taxi GPS traces are collected from a Beijing taxi company from April to August 2012. From the taxi GPS data, we extract the information of each trip, which includes the pick-up location, pick-up time, drop-off location, drop-off time, trip distance, trip speed, driving direction, trip cost, and passenger number.
• *Bus Traces Data.* As two important types of public transit, buses are cheaper with acceptable speeds than taxis that are expensive with faster speed. In urban areas, massive residents choose buses. We have collected Beijing bus trip data through the records of the bus smart card system. Each trip consists of the card id, time stamp, expense, balance, route name, and pick-up and drop-off stop information (names, longitudes, and latitudes).
• *Point of Interest Data.* A point of interest, or POI, is a specific point location that someone may find useful or interesting. We have collected a comprehensive dataset of POI information of Beijing from Dianping and Dajie, including POI name, POI category, latitude, and longitude. The POI categories include catering, shopping, living, sports and leisure, health care, accommodation, scenic spots, business residential, government agencies, science and education, transport facilities, finance and insurance, corporate, and public facilities.
• *Mobile Check-in Data.* Location-based social networks (LBSNs), such as Foursquare, Yelp, and Facebook places, have attracted millions of users to share their digital footprints and opinions with their friends and have enabled us to collect check-ins from mobile apps. Each check-in event typically includes POI name, POI category, address, longitude and latitude, textual comments, and geographic tags. We have collected Beijing check-in data from Weibo, a Chinese version of twitters. It contains 2,762,128 check-ins in 5,874 venues.

Table 2 shows the statistics of five data sources.

**TABLE 2:** Statistics of the experimental data.

Data source	Properties	Statistics
Taxi GPS	Number of taxis	13,597
	Effective days	92
	Time period	April–August 2012
	Number of trips	8,202,012
	Number of GPS points	111,602
	Total distance (km)	61,269,029
Bus/subway traces	Number of bus/subway stops	9,810
	Time period	August 2012–May 2013
	Number of car holders	300,250
	Number of trips	1,730,000
Mobile check-ins	Number of check-in POIs	5,874
	Number of check-in events	2,762,128
	Number of POI categories	9
	Time period	01/2012-12/2012
POIs	Number of business POIs	328,668
	Positions (longitude and altitude)	328,668
Residential communities	Number of real estates	2,990
	Size of bounding box (km)	40*40

### Baseline Algorithms

To show the effectiveness of our method, we compare our method against the following algorithms.

• RankNet (Burges et al., 2005): It is a combination of a simple probabilistic cost function and using gradient descent methods for learning ranking functions, using a neural network to model the underlying ranking function.
• ListNet (Cao et al., 2007): It is a listwise ranking model with permutation top-k ranking likelihood as objective function. ListNet introduces two probability models, respectively, referred to as permutation probability and top-k probability, to define a listwise loss function for learning. Neural network and gradient descent are then employed as model and algorithm in the learning method.
• Coordinate Ascent (Dang and Croft, 2010): It uses a loss function called the domination loss. Coordinate ascent extends the loss by incorporating margin requirements over pairs of instances and enables the usage of multivalued feedback. Coordinate ascent devises a simple yet effective coordinate descent algorithm that is guaranteed to converge to the unique optimal solution.
• Random Forests (Jiang, 2011): It is a ranking strategy through learning the predictions from an ensemble of random trees.

In the experiments, we utilize RTree^1^ to index geographic items (i.e., taxi and bus trajectories) and extract the defined features. We use Jieba,^2^ which is a Chinese/English text segmentation module to segment words and extract tags.

For traditional LTR algorithms, we use RankLib.^3^ We set the number of training epochs to 100, the number of hidden layers to 1, the number of hidden nodes per layer to 10, and the learning rate to 0.00005 for RankNet. We set the number of iterations to 300 and the number of threshold candidates to 10 for RankBoost. We set number of random restarts to 5, the number of iterations to search in each dimension to 25, and tolerance to 0.001 for Coordinate Ascent. We set the number of bags to 300, the number of leaves to 10, the number of threshold candidates to 256, the number of leaves for each tree to 100, and the learning rate to 0.1 for Random Forest. We set *a* to 0.001, *b* to 0.001, and $\sigma^{2}$ to 1,000 for our model.

All the codes are implemented in Python, including modeling, feature extraction, and visualization. All codes can be downloaded *via* the link.^4^ And all the evaluations are performed on a x64 machine with i7 2.50 GHz Intel CPU (with four cores) and 16 GB RAM. The operation system is OS X EI Capitan.

### Evaluation Metrics

To evaluate the effectiveness of the proposed model, we use the following metrics.

**•** Normalized Discounted Cumulative Gain (NDCG@N).

The discounted cumulative gain (DCG@N) is given by

$$DCG\left[n\right]=\left\{ \begin{array}{l} rel_{n}\;\;\;\;\;\;\;\;\;\;\;\;\;\;\;\;\;\;\;\;\;\;\;\;\;\;\;\;\;\;\;\;\;\;\;if\;n=1\\ DCG\left[n-1\right]+\frac{rel_{n}}{log_{2}n},\;if\;n>=2 \end{array}\right.,$$ (26)

where $rel_{n}$ denotes the vibrancy grade level of the *n*-th community, defined in Eq. 2. Later, given the ideal discounted cumulative gain $DCG^{\prime }$, NDCG at the n-th position can be computed as

$$NDCG\left[n\right]=\frac{DCG\left[n\right]}{DCG^{\text{′}}\left[n\right]}.$$ (27)

The larger NDCG@N is, the higher the top-N ranking accuracy the classifier has.

• Kendall’s Tau coefficient.

Kendall’s Tau coefficient (or Tau for short) measures the overall ranking accuracy. Let us assume that each community *i* is associated with a benchmark vibrancy $y_{i}$ and a predicted vibrancy score $f_{i}$. Then, for a community pair <i,j>, <i,j> is said to be concordant, if both $y_{i}>y_{j}$ and $f_{i}>f_{j}$ or if both $y_{i}<y_{j}$ and $f_{i}<f_{j}$. Also, <i,j> is said to be discordant, if both $y_{i}<y_{j}$ and $f_{i}>f_{j}$ or if both $y_{i}<y_{j}$ and $f_{i}>f_{j}$. Tau is given by

$$\text{Tau}=\frac{{\#}_{conc}-{\#}_{disc}}{{\#}_{conc}+{\#}_{disc}},$$ (28)

where ${\#}_{conc}$ denotes the concordant pairs and ${\#}_{disc}$ denotes the discordant pairs.

• Recall.

Since we use a six-level rating system ( 5>4>3>2>1>0) instead of the binary rating, we treat the rating ≥ 5 as “highly vibrant” and the rating < 5 as “fairly vibrant.” Given a top-N estate list $E_{N}$ sorted in a descending order of the prediction values, the recall is defined as

$$\text{Recall}@N=\frac{\left|E_{N}\cap E_{\geq 5}\right|}{\left|E_{\geq 5}\right|},$$ (29)

where $E_{\geq 5}$ are the estates whose ratings are greater or equal to 5.

### Analysis of Scoring Community Vibrancy

**FIGURE 3:**
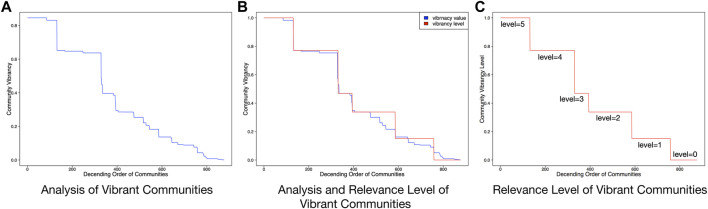
Analysis of urban vibrancy based on the proposed metric.

We calculate the vibrancy scores of residential communities in the dataset based on the proposed metric Eq. 2. After that, all the communities are sorted in a descending order in terms of vibrancy scores, as shown in Figure 3A. We can observe that there are some fault ages on the curve, where the vibrancy scores of some communities significantly increase, whereas the vibrancy scores of many communities remain stable. To prepare the grade levels of community vibrancy for our ranking framework, we utilize these inflection points. First, we identify five inflection points in the curve, which, respectively, denote the vibrancy scores of 0.7713, 0.4685, 0.3375, 0.1506, 0.0523, and 0.7713. The five inflection points split the curve into six segments. After that, we assign six-level ratings to each segment as its ranking relevance label, for instance, 5, 4, 3, 2, 1, and 0, respectively, in a descending order based on the vibrancy scores. As a result, we obtain six rating levels for the ranking process, as shown in Figures 3B,C.

The curve in Figure 3A shows that the distribution of the community vibrancy scores complies with a power law distribution, indicating only a small number of residential communities are highly vibrant, and most communities are around the mean value of the vibrancy scores. This observation is consistent with our common sense about our world: Most people are middle class and only a small number are rich. The six rating levels are shown in Figures 3B,C, which visualizes the distribution of the six vibrancy levels of all the communities.

### Correlation Analysis of Features

**FIGURE 4:**
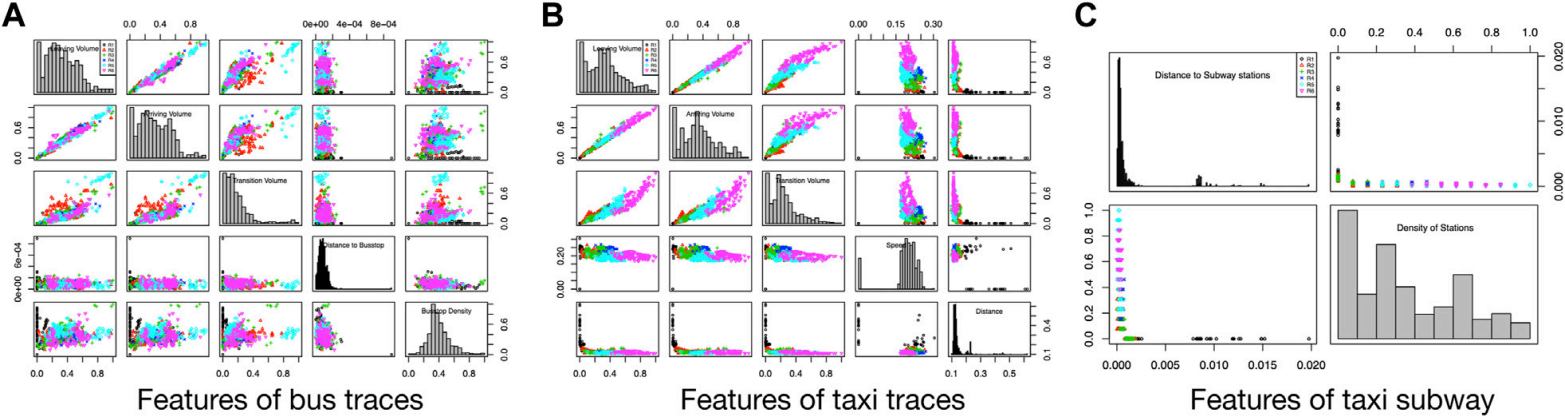
Feature correlation analysis of bus traces, taxi traces, and subways.

We provide a visualization analysis to validate the correlation between the extracted features and the vibrancy scores of communities. We use the scatter plot matrix for correlation analysis. Each non-diagonal chart in a scatter plot matrix shows the correlation between a pair of features whose feature names are listed in the corresponding diagonal charts. Given a set of N features, there are N-choose-two pairs of features, and thus the same numbers of scatter plots. The dots represent the communities and their colors represent the levels of vibrancy values. For readability, we use R6>R5>R4>R3>R2>R1 (symbol) to represent 5>4>3>2>1>0 (number) in Figure 4. For detailed quantitative results, refer to Table 3.

**TABLE 3:** Feature correlation analysis of bus traces, taxi traces, and subways.

	R6	R5	R4	R3	R2	R1
Inflow of bus	0.63	0.85	0.36	0.11	0.56	0.17
Outflow of bus	0.59	0.88	0.34	0.57	0.49	0.24
Intra-flow of bus	0.67	0.92	0.31	0.37	0.28	0.05
Distance to bus stop	0.21	0.57	0.86	0.74	0.42	0.45
Bus stop density	0.14	0.09	0.88	0.18	0.50	0.65
Inflow of taxi	0.89	0.73	0.79	0.59	0.14	0.51
Outflow of taxi	0.94	0.16	0.41	0.12	0.61	0.05
Intra-flow of taxi	0.85	0.62	0.84	0.49	0.42	0.27
Speed of taxi	0.92	0.13	0.26	0.01	0.65	0.88
Traveling distance of taxi	0.89	0.36	0.51	0.33	0.80	0.25
Distance to subway station	0.89	0.63	0.47	0.10	0.07	0.01
Subway station density	0.93	0.82	0.45	0.19	0.08	0.02

In Figure 4A, we present the correlation between bus trace features (inflow interaction, outflow interaction, intra-flow interaction, distance to bus stops, and the density of) and vibrancy values of communities. As can be seen, the R5 communities tend to appear at the top right corner of all the non-diagonal charts. However, the R6 communities appear at the middle of the figure. This implies that the bus is the major transportation for common communities, while people tend to visit top vibrant and high-end communities by other kinds of vehicles.

In Figure 4B, we show the positive correlation between the taxi inflow, outflow, and intra-flow volumes of communities and vibrancy values. This shows that the taxi is an important transportation to visit vibrant communities, which is consistent with the observation of buses in Figure 4A. However, the commute distances of taxis have a negative correlation with the vibrancy scores. In other words, the shorter the commute distances of taxis are, the higher the vibrancy scores of residential communities are. A potential interpretation of this observation is that since taxis are valued by white-collar and business people, the destinations of taxi trajectories usually are important places (i.e., conference centers, business hotels, companies, and government organizations). If the commute distance of taxis is shorter, the targeted neighborhood is closer to these important places.

In Figure 4C, we show the power law correlation between the community vibrancy scores and the subway-related features, including the distance to the subway stations and the density of the subway stations. We can obtain the observation similar to Figure 4A that subways are not the most important transportation for visiting top vibrant communities. Based on the observations in Figures 4A,B, we can find that the public transportation (i.e., bus and subway) has huge effects on the communities of R4 and R5. However, the influence of the public transportation on top vibrant communities is small. The taxi-related features show nearly a positive linear relation with the community vibrancy scores, especially for top vibrant communities (R6). There may be an explanation that if a community is very vibrant, the cost spent on transportation is likely to be high. As known to all, public transportations are relatively slow but cheap. Taxis are expensive but fast. Therefore, the high-consumption group (like white-collar and business people) who can afford taxis are more in favor of taxis.

In summary, the visualization results show the correctness of our intuitions about defining and extracting discriminative features.

### Examining the Importance of Urban Geography and Human Mobility Features

**FIGURE 5:**
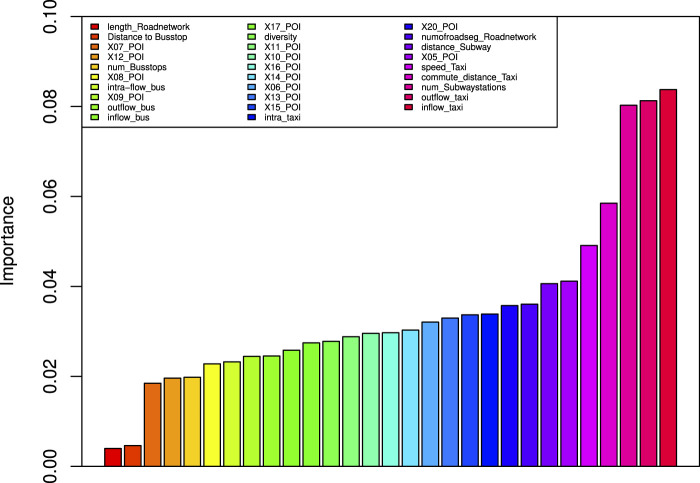
Feature importances based on information gain.

We measure the information gain of each feature described in the section *Discovering Patterns of Vibrant Communities* to understand the importance of the spatial and mobility patterns in community vibrancy. Specifically, we calculate the information gain of each feature for each vibrancy levels (i.e., 5>4>3>2>1>0) across our data. Figure 5 shows the results of the information gain analysis for a decision tree classifier.

We have some interesting observations from Figure 5:

• Taxi-related features including inflow, outflow, commute distance, and speed are top ranked as 0.084, 0.081, 0.058, and 0.049, respectively. Surprisingly, the intra-flow of taxi is ranked as 0.034 in the middle of the list. This conforms with our common sense that the human mobility across communities encodes both specific trip purposes and the destinations that can meet people’s demands. That is exactly why the vibrant communities can attract people. However, the mobility within communities cannot show the sign explicitly.
• The information gain of POI-related features distributes in the range of the list. The highest is 0.041, while the lowest is 0.018. The reason for such big differences can be that different POI categories always have different functionalities. Some POIs, like shopping and restaurants, are popular to people and can provide the recreation and entertainment functionality, while some POIs, like vehicle services, would not appear too many in our daily life. Therefore, specific POI categories may contribute a lot to the community vibrancy but some may not.
• The public transportation–related features including the distance to bus stops, the number of bus stops, the intra-flow of buses, the outflow of buses, the distance to subway stations, and the number of subway stations are ranked at 0.005, 0.020, 0.023, 0.025, 0.034, 0.041, and 0.080, respectively. Moreover, the subway-related features are more important than the bus-related features. There is a possible explanation that the subway is much more rapid than the bus and we also do not need to worry about the traffic jam on the subway. In this case, the more rapid and convenient subway outweighs.
• For road network–related features, that is, the length of road networks and the number of intersections, the information gain value is 0.004 and 0.036. We need to notice that the information gain of the number of intersections nearly catch up with taxi-related features. This is because more intersections mean that it is more likely to take a taxi. Convenient transportation facilities in the vibrant communities always attract many people to visit.

### Model Performance Comparison

We compare the performance of our method with four baseline algorithms in terms of Tau and NDCG.

**FIGURE 6:**
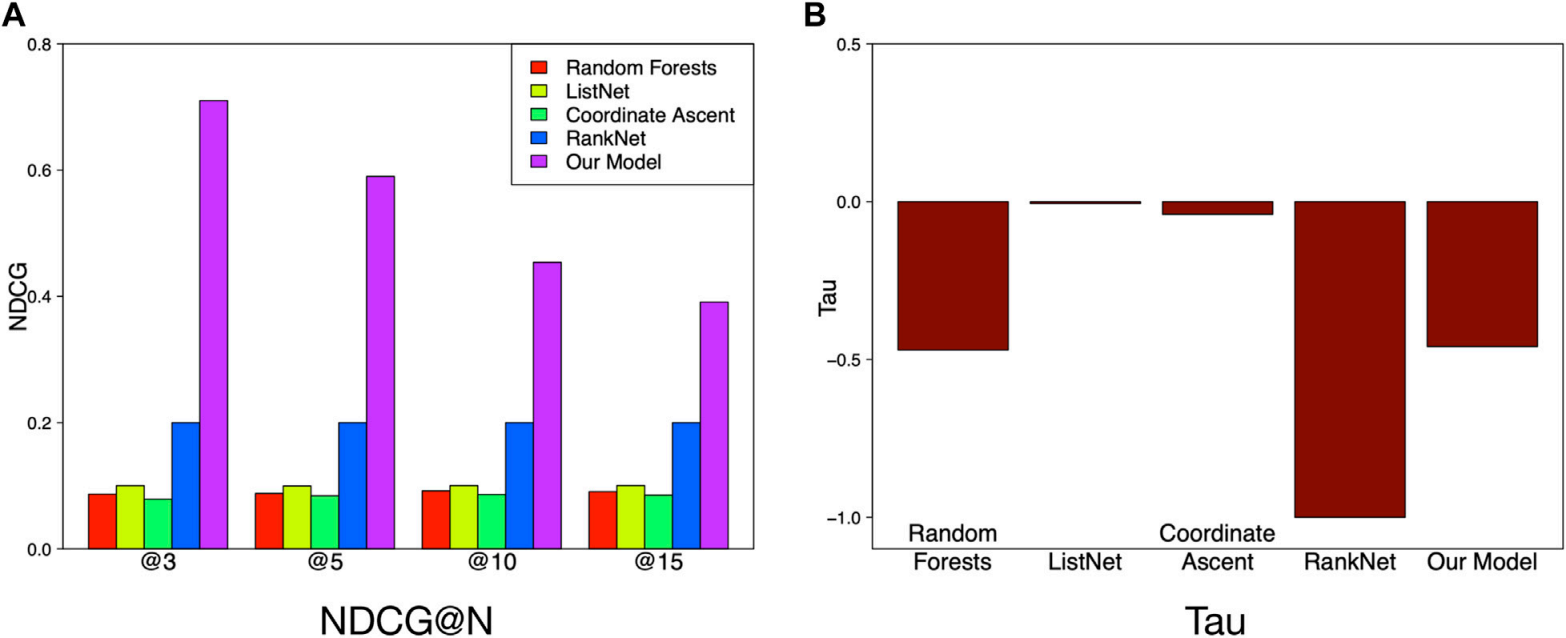
Performance comparison between models.

In Table 4, we list details of performance of different models. Our method achieves 0.6081 NDCG@3, 0.5283 NDCG@5, 0.3736 NDCG@10, and 0.3314 NDCG@15, which obviously outperforms the baseline algorithms with a significant margin. Our method fuses sparsity regularization and pairwise ranking objective and offers an increase in comparison to RankNet which has the best performance in baseline algorithms, as shown in Figure 6.

**TABLE 4:** Performance comparison of our approach and baselines.

	Random Forests	ListNet	Coordinate Ascent	RankNet	Our model
NDCG@3	0.0867	0.1002	0.0788	0.2	0.7103
NDCG@5	0.0879	0.0997	0.0841	0.2	0.5897
NDCG@10	0.0919	0.1003	0.0861	0.2	0.4544
NDCG@15	0.0907	0.1004	0.0852	0.2	0.3908
Tau	−1.0	−0.0401	−0.0616	−0.4699	−0.4594

This observation validates the superiority of our method when considering many intercorrelative features with confounders. Moreover, the effectiveness of considering both sparsity regularization and ranking accuracy is proved.

With respect to the overall ranking, our method achieves the highest Tau (0.6137). Surprisingly, all the baselines perform badly on Tau where values of Tau are all negative. The observation indicates that the number of concordant pairs is slightly less than the number of discordant pairs, which demonstrates the lower accuracies of the baseline algorithms on the whole ranking list. However, our method achieves a balanced performance in both top-k and overall ranking.

Another fact we draw from Figure 6 is that the NDCG of our model increases with N getting small, which indicates that the ranking performance of our model does well in the top-k ranking task, especially for the very top part.

### Feature Performance Comparison

We evaluate the performances of different features segmented from two angles. The feature performance is evaluated in terms of NDCG@N, Recall@N, and Tau, respectively.

**•** Evaluation on features of different categories.

**FIGURE 7:**
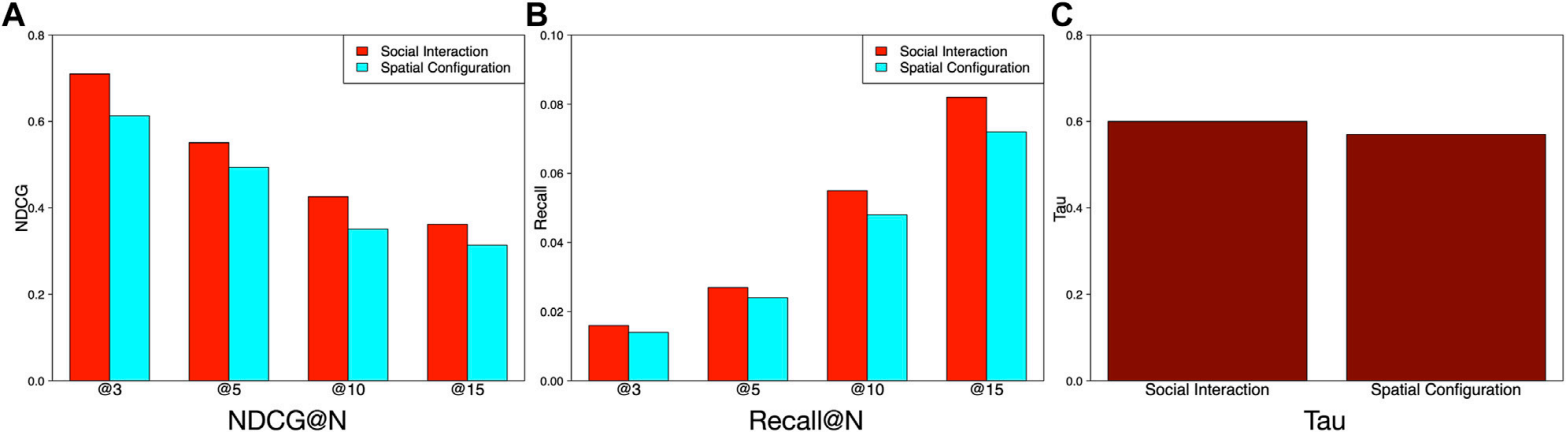
Comparison of feature performance based on different states.

At the beginning of the article, we emphasize that the vibrancy of community is valued in terms of spatial configurations and social interactions. The difference between spatial configurations and social interactions is that spatial configuration represents the static state of a community, implying the geographical representations and distributions of static geographic items, like POIs and bus stops, whereas social interactions represent the dynamics of a community, showing the mobility patterns of mobile objects, like taxis and buses. Therefore, we split features into these two categories. As shown in Figure 7A, compared to spatial configuration features, social interaction features perform better. This observation shows that dynamic features contribute more to the ranking accuracy of our model. It is very necessary to study the social interaction features to further explore more useful dynamic patterns for improving ranking performances. Besides, Figures 7B,C also provide other evidences to validate the better performance of the social interaction features compared with the spatial configuration features in terms of Recall@N and Tau.

• Evaluation on features of different data sources.

**FIGURE 8:**
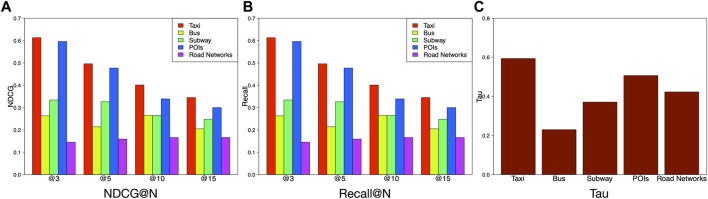
Comparison of feature performance based on data sources.

Aside from studying the categories of features, we also study the performances of different data sources as we have collected data from taxi GPS trajectories, bus GPS trajectories, road networks, and POIs. Here, we segment the extracted features in terms of different data sources and investigate which source is more effective for ranking urban vibrancy. Figure 8A shows the taxi and POI–related features contribute most to the accuracy of the proposed model, while the road network–related features contribute the least to the model accuracy. Moreover, taxi data and POI data are the two major sources to represent the social interactions and spatial configurations, respectively. This observation is consistent with the result in Figures 7, 8B which show taxis data performance is the best among all the data sources. The following are bus, subway, POIs, and road networks, respectively. As for Tau, the performances of different data sources are ranked in a descending order as taxi > POIs > road networks > subway > bus. Overall, taxi-related data are the most useful source to construct effective features. Besides, when we examine the performances of all kinds of transportation-related data, taxi > subway > bus. Here is a possible explanation. Bus is the most common type for commuting. Also, bus and subway are for massive people traveling in a certain planed trajectories based on schedule. However, taxis are for personal usage, making the range of traditional zone unlimited. Therefore, we can dig more information from taxi-related features.

• Evaluation on features of different radius distances.

**FIGURE 9:**
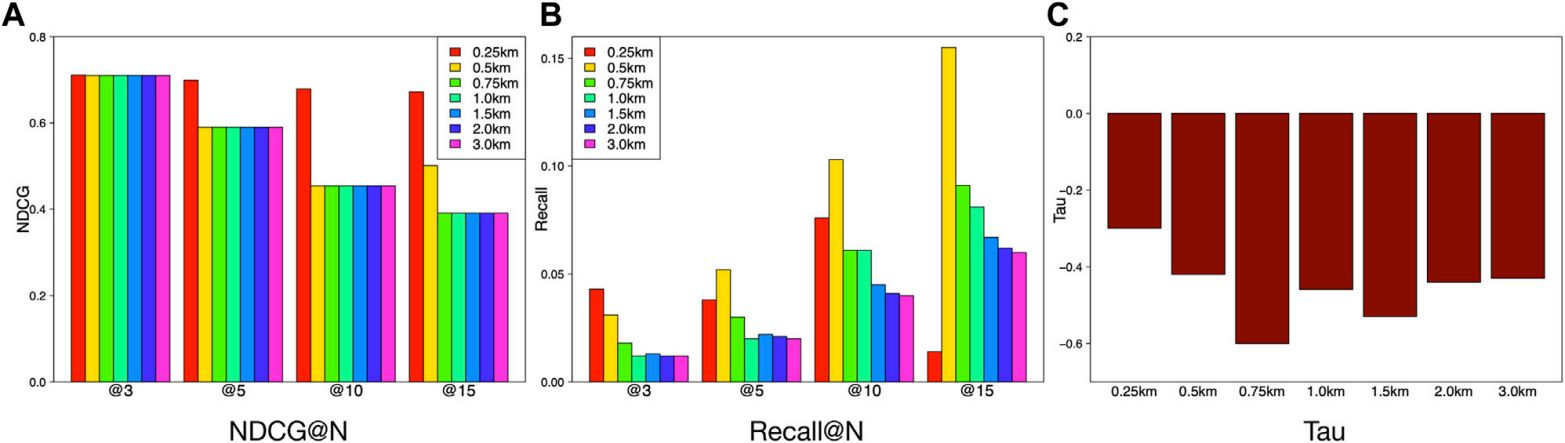
Comparison of feature performance based on different radius distance.

We segment the features in terms of different radius of communities and investigate the proper radius of neighborhoods for ranking community vibrancy. Figure 9 shows the performance comparisons of the feature sets of different radius distances (i.e., 0.25, 0.5, 0.75, 1, 1.5, 2, and 3 km). We observe that the radius distance of neighborhood can affect the ranking performance. Figure 9A shows that the NDCGs for 0.5, 0.75, 1, 1.5, 2.0, and 3 km are almost same, while the radius of 0.25 km shows a slightly higher NDCG. However, the high NDCGs of 0.5–3 *km* are used to consistently validate the superiority of our model. For the recall performances in Figure 9B, we can obtain an interesting observation that there is a descending trend when the radius is getting larger. This may be due to the fact that more data are available when the community radius is larger. Abundant data result in poor generalization of a model and lead to the descending trend of the ranking accuracy. Figure 9C implies that the Tau values vary slightly in the interval of [−0.3,−0.6] when the radius of communities drops (0.25km,0.5km,0.75km,1km,1.5km,2.0km,3km). This reflects the robustness of our method from another perspective.

Based on the above analysis, we should not set the radius of communities too small (i.e., 0.25km) because of the information limitation. On the other hand, too large value of radius is useless due to the stability of the ranking performance. Therefore, we set the radius as 1 *km* in this study.

## Related Work

### Urban Planning

Researchers have developed conceptual and empirical measurements on urban vibrancy from different aspects. The first aspect is density. The work in Glaeser et al. (2001) pointed out modern cities will be consumer-centric rather than production-centric; the future of cities depends on the demand for urban *density*. Couture *et al.* found that high-density areas benefit residents in terms of more social interaction and diverse consumption opportunities, and people are willing to pay higher rents and transportation costs for high-density places (Couture, 2013). The second aspect is diversity. Farber *et al.* found that proper urban structure leads to spatial concentration of residents and diversity of products and services (Farber and Li, 2013). Talen *et al.* found that mixed land uses can encourage workability and foster social interaction (Talen, 1999). Malizia *et al.* found that vibrant communities are usually compact, dense, and accessible with diverse land uses (Malizia and Song, 2014). Neutens *et al.* found that high-density and mixed land uses can benefit quality social interaction and enhance vibrancy (Neutens et al., 2013). The third aspect is human-related dynamic factors. Dougal *et al.* argued urban vibrancy should be measured by dynamic human-dependent factors that vary over time (Dougal et al., 2015). For example, Farber and Li (2013) proposed social interaction potential as a measurement; Audretsch et al. (2003) proposed the knowledge diffusion among workers as a metric; Jaffe et al. (1993) measured vibrancy with technology spillovers between neighboring firms; Glaeser et al. (2001) used consumption externalities between its residents as a metric; and the work by Dougal et al. (2015) devised firm investment opportunities as a metric. In summary, prior studies found that 1) urban vibrancy is nearly always related to density and diversity in terms of both static geographical and dynamic human-related factors; and 2) urban vibrancy is complex and should include density, diversity, and human activities.

### Urban Computing With Geography and Mobility

Urban computing (Zheng et al., 2014) is a process of acquisition, integration, and analysis of urban data (e.g., sensors, devices, vehicles, buildings, and human) to tackle the major issues that cities face. Our work also has a connection with mining mobile, geography, and mobility data to tackle issues in urban space. Tseng et al. mine the behavior patterns from mobile sensor data to enhance system performance (Tseng and Lin, 2006). The work by Ceci et al. (2007) identifies emerging patterns with multirelational approach from spatial data. Liu et al. detect spatiotemporal causality of outliers in traffic data (Liu et al., 2011). Yuan et al. discover regional functions of a city using POIs and taxi traces (Yuan et al., 2012). Heierman et al. mine the device usage patterns of homeowners for smart houses (Heierman and Cook, 2003). The study by Karamshuk et al. (2013) selects the optimal sites for retail stores by mining Foursquare data. Zheng et al. (2014) mine the driving route for end users by considering the physical feature of a route, traffic flow, and driving behavior.

### Learning-to-Rank

Our work can be categorized into learning-to-rank (LTR), which includes pointwise, pairwise, and listwise approaches (Li, 2011). The pointwise methods (Li, 2011) reduce the LTR task to a regression problem: given a single query–document pair, it predicts its score. The pairwise methods reduce the LTR task to a classification problem. The goal of the pairwise ranking is to learn a binary classifier to identify the better document in a given document pair by minimizing the average number of inversions in ranking, for example, RankNet (Burges et al., 2005), RankBoost (Freund et al., 2003), RankSVM (Herbrich et al., 2000), and LambdaRank (Burges et al., 2007). The listwise methods optimize a ranking loss metric over lists instead of document pairs (Xia et al., 2008). For instance, H. Li et al. propose AdaRank (Xu and Li, 2007) and ListNet (Cao et al., 2007) and Burges et al. propose LambdaMART (Burges, 2010). The recent work by Agarwal et al. (2012) and Agrawal et al. (2006) further studied multifaceted ranking and context-sensitive ranking. The work by Rendle et al. (2009), Weng and Lin (2011), and Gantner et al. (2012) provide full Bayesian explanations and optimize the posterior of pointwise, pairwise, and listwise ranking models, respectively. The study by Shi et al. (2013) unifies both rating error and ranking error as objective function to enhance top-k recommendation. More recent work (Lai et al., 2013) further learns the ranking model which is constrained to be with only a few nonzero coefficients using L1 constraint and proposes a learning algorithm from the primal dual perspective.

## Conclusion Remarks

In this article, we aimed to measure urban vibrancy by examining spatial configuration and social interaction of communities with Big Crowdsourced Geotagged Data. We proposed a fused scoring framework, combining diversity and density of consumer activities with F-1 score. We extracted features to represent spatial configuration and social interaction, respectively. To learn vibrancy values based on the proposed scoring framework, we designed a sparse ranking model which is mutually enhanced by simultaneously conducting feature selection and maximizing communities’ vibrancy ranking accuracy. Finally, the experimental results with BCGD demonstrate the competitive effectiveness of both extracted features and learning models. With the high accuracy ranking prediction, we explore the potential to use BCGD for providing useful strategies for governments on urban planning. On the other hand, higher vibrancy leads to more consumers and the high quantity of consumers enhance vibrant communities, which invents a virtuous cycle for the development of cities.

## Data Availability

The data analyzed in this study are subject to the following licenses/restrictions: Data belong to Microsoft. Requests to access these datasets should be directed to yanjie.fu@ucf.edu.

## Footnotes

1
https://pypi.python.org/pypi/Rtree/

2
https://github.com/fxsjy/jieba

3
http://sourceforge.net/p/lemur/wiki/RankLib/

4
https://www.dropbox.com/s/tyamms9625aivtk/code.py?dl=0
